# Ampicillin-Ester Bonded Branched Polymers: Characterization, Cyto-, Genotoxicity and Controlled Drug-Release Behaviour

**DOI:** 10.3390/molecules19067543

**Published:** 2014-06-06

**Authors:** Ewa Oledzka, Marcin Sobczak, Grzegorz Nalecz-Jawecki, Agata Skrzypczak, Waclaw Kolodziejski

**Affiliations:** 1 Department of Inorganic and Analytical Chemistry, Medical University of Warsaw, Faculty of Pharmacy, Banacha 1, Warsaw 02-097, Poland; E-Mails: marcin.sobczak@wp.pl (M.S.); waclaw.kolodziejski@wum.edu.pl (W.K.); 2 Department of Environmental Health Science, Medical University of Warsaw, Faculty of Pharmacy, Banacha 1, Warsaw 02-097, Poland; E-Mails: gnalecz@wum.edu.pl (G.N.-J.); agata.skrzypczak@wum.edu.pl (A.S.)

**Keywords:** controlled release, drug delivery system, ampicillin, cyto- and genotoxicity macromolecular conjugate

## Abstract

The development and characterization of novel macromolecular conjugates of ampicillin using branched biodegradable polymers has been described in this study. The conjugates have been prepared coupling the β-lactam antibiotic with branched polymer matrices based on the natural oligopeptide core. The cyto- and genotoxicity of the synthesized polymers were evaluated with a bacterial luminescence test, two protozoan assays and *Salmonella typhimurium* TA1535. The presence of a newly formed covalent bond between the drug and the polymer matrices was confirmed by ^1^H-NMR and FTIR studies. A drug content (15.6 and 10.2 mole %) in the macromolecular conjugates has been determined. The obtained macromolecular products have been subjected to further *in vitro* release studies. The total percentage of ampicillin released after 21 days of incubation was nearly 60% and 14% and this resulted from the different physicochemical properties of the polymeric matrices. This is the first report on the application of branched biodegradable polymeric matrices for the covalent conjugation of ampicillin. The obtained results showed that the synthesized macromolecular drug-conjugates might slowly release the active drug molecule and improve the pharmacokinetics of ampicillin.

## 1. Introduction

β-Lactam antibiotics constitute a broad class of antimicrobial agents, applied for systemic therapy and gastric or intestinal infections [1]. The presence of the β-lactam ring in their structure is responsible for their antimicrobial activity. Ampicillin ((2*S*,5*R*,6*R*)-6-([(2*R*)-2-amino-2-phenylacetyl]amino)-3,3-dimethyl-7-oxo-4-thia-1-azabicyclo[3.2.0]heptane-2-carboxylic acid) is a semi-synthetic penicillin belonging to the β-lactam antibiotic family which has found widespread use due to its broad spectrum of activity. Ampicillin is absorbed only partially after oral administration and ordinarily about 50% of active compound is extracted in urine [1]. Ampicillin therapy is often accompanied by some gastrointestinal complications such as diarrhoea, vomiting and nausea because of the low solubility of the drug in lipid membranes [2]. Furthermore, ampicillin like other β-lactams, crosses the cell membrane very slowly and it’s unable to reach therapeutic concentrations at the site of infection [3,4].

Drugs administrated systemically are absorbed into the blood stream and distributed throughout the host patient via the circulatory system, which can result in bacterial resistance [5]. When administrated locally, they limit the adverse effects of systemic administration and a higher concentration of drug is reaching the target site [6]. Therefore, there is a growing need to provide adequate drug concentrations at the site of action and a means of maintaining that level for a long enough period to allow the agent to act [7].

The first usage of biomaterials in medicine has provided some important complications (infections and deficient tissue-integration) in humans. Implant-associated infections account for nearly 50% of the estimated 2-million nosocomial infections in the United States per year, and infection rates up to 100% are reported for certain implants, like external fixation pins [8,9,10]. Treatment of these infections is associated with high complication rates and places an enormous burden on both the patient and healthcare providers; prolonged hospital stay, increased morbidity and mortality and serious economic consequences [11]. Hence, the risk of infection might be reduced by the implementation of the polymeric implants formed by covalent conjugation of antibacterial agent to the biodegradable or bioresorbable polymers.

Polymer-drug conjugates are nano-sized hybrid constructs that covalently combine a bioactive agent with a polymer to ensure not only its efficient delivery to the required intracellular compartment, but also its availability within a specific period of time [12]. The use of polymer-drug conjugates in combination therapy is seen as an important opportunity to enhance disease response rates. The macromolecular prodrugs comprise a minimum of three components, as shown a natural or synthetic polymeric carrier, a biodegradable polymer-drug linkage (often a peptidyl or ester linkage) and a bioactive agent [12]. It is assumed that the therapeutic effectiveness of a polymer prodrug is governed by the chemical or biochemical release of the drug molecule. The controlled slow release of pharmacologically active agent in the body might be achieved from prodrugs, which can be considered as a special type of implantable drug delivery system from which drug release is accomplished by the cleavage of chemical bond [13].

Biodegradable or bioresorbable polymers belong to a class of important and desirable biomaterials because of their wide applications in the biomedical fields; including tissue engineering, controlled drug delivery and gene therapy [14]. Aliphatic polyesters, such as polylactide (PLA), and poly(ε-caprolactone) (PCL) are the most commonly used due to their good biocompatibility, biodegradability, low immunogenicity and suitable mechanical properties [15].

Recently, a few approaches using local ampicillin polymeric delivery system materials have been introduced, which allows the therapeutic agent to be targeted to the disease site with minimal systemic effects. Patel *et al.* prepared the polymeric matrices of poly(styrene-co-maleic anhydride) on which surfaces ampicillin was bound by chemical bonding in an organic medium [13]. PCL electrospun fibers containing ampicillin sodium salt have been produced and twisted into nanofibers yarns by Liu and coworkers [16]. Their results indicated that the electrospun nanofibers yarns can have a great potential to be used for biomaterials to decrease surgical site infection rates. Ampicillin was also reacted with expanded polytetrafluoroethylene surfaces resulting in the formation of antimicrobial surfaces active against both Gram-positive and Gram-negative bacteria [17], while sodium ampicillin was incorporated in a hybrid organic-inorganic material of the PCL and titanium to verify the effect as a local controlled drug delivery system [18]. However, to the best of our knowledge, there is no report on the covalent conjugation of ampicillin to PCL and PLA for the creation of branched implantable drug delivery carriers.

In our previous paper, natural arginine-6-oligomer has been effectively engaged as an initiator of the ring opening polymerization (ROP) of L-Lactide (LA) and ε-caprolactone (CL) for the synthesis of the branched biodegradable polymers, which exhibited therapeutic properties adequate for the drug carrier applications [19]. The physicochemical characterization of the obtained polymers has been confirmed by various applied techniques. This report is the sequel to our previous work, that is ampicillin has been covalently (ester) bonded to the obtained branched polymers using the DCC/DMAP coupling method (DCC = *N*,*N*'-dicyclohexylcarbodiimide, DMAP = 4 (dimethylamino)pyridine). *In vitro* release rate of the drug from the biodegradable macromolecular conjugates was analysed. Furthermore, geno- (the *umu*-test) and cytotoxicity tests of the received branched polymers were evaluated with a bacterial luminescence test, two protozoan assays and *Salmonella typhimurium* TA1535. Although cell culture assays with mammalian cells are currently the most popular *in vitro* tests for evaluating acute toxicity, other techniques are gaining prominence. They involve the use of bacteria and lower *Eucaryotes* [20] Luminescence assay with *Vibrio fischeri* has become broadly used as a fast and reliable preliminary test for risk assessment [21]. The use of protists, especially protozoa, as non-animal has the potential of reducing, refining and replacing *in vivo* testing (3R concept). *Tetrahymena* is the most widely used ciliated protozoan [22].

## 2. Results and Discussion

Work on the synthesis and characterization of macromolecular (polyester, polyurethane, poly(amideurethane)s) conjugates of fluoroquinolones, anti-cancer and anti-inflammatory drugs have been initiated in our laboratory in 2006 [23,24,25,26]. This work is a continuation of our research group’s trend extended now to the branched polyester conjugates of ampicillin. The branched macromolecular carriers composed of biodegradable PCL and PLLA with incorporated natural 6AR have previously been synthesized and structurally characterized [19]. According to the pharmacopoeia standards for materials used for biomedical purposes, *inter alia* toxicity, the received products (6AR/PCL and 6AR/PLLA) have been subjected to the cyto- and genotoxicity assays, by applying the luminescent bacteria *V. fischeri*, two ciliated protozoa, *S. ambiguum*, *T. thermophila* (Table 1), and *Salmonella typhimurium*. It has been found that the obtained polymers are not cytotoxic to any of the test bacterial and protozoal bionts and thereby they are adequate for biomedical applications owing to the fact, that a material is considered to be toxic, when the percent of a toxic effect (PE) is higher than 20 [27]. Concerning genotoxicity results, no tested compounds showed genotoxic potential for *Salmonella typhimurium* TA1535 in the *umu*-test regardless of the s9 fraction used. The activity of β-galactosidase remained at the level of negative control when either the polymers’ extracts or the material pieces were investigated.

**Table 1:** The cytotoxicity of the obtained polymer samples.

	Spirotox (24 h-PE ^a^)		Microtox^®^ (15 min-PE ^a^)		Protoxkit F™ (24 h-PE ^a^)	
Concentration (1 mg/mL)	0.5	1	0.5	1	0.5	1
6AR/PCL	0	0	0	0	0	0
6AR/PLLA	0	0	0	0	0	0

^a^ Percent of toxic effect.

**Scheme 1:**
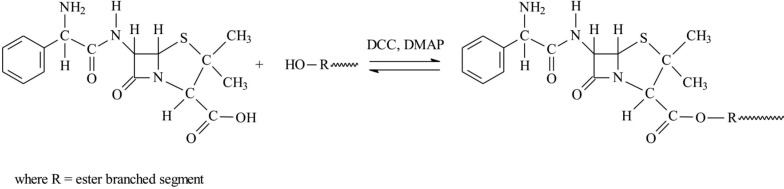
Synthesis of the branched polyester conjugates of ampicillin.

Ester bonds were formed between the surface hydroxyl groups of the branched 6AR/PCL or 6AR/PLLA and the carboxyl group of ampicillin using an active ester method involving DCC and DMAP (Scheme 1).

**Figure 1:**
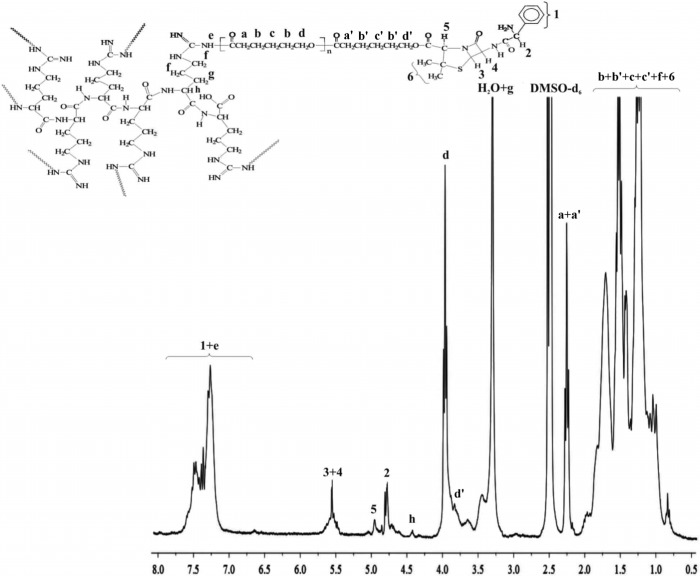
^1^H-NMR spectrum of the 6AR/PCL/ampicillin conjugate.

**Figure 2:**
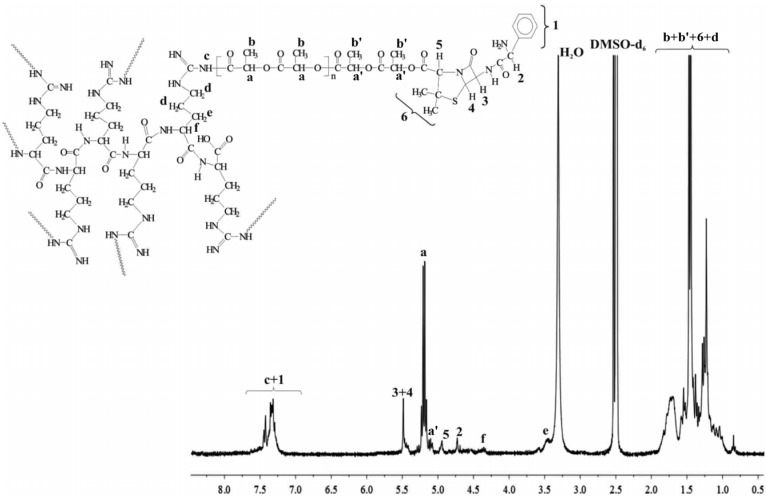
^1^H-NMR spectrum of the 6AR/PLLA/ampicillin conjugate.

Ampicillin was reacted with the branched polymeric samples in 1.5 molar ratio. In view of the number of surface hydroxyl groups of the polymers (14 for 6AR/PLLA and 16 for 6AR/PCL) 21 and 24 M equivalent of ampicillin was added to the polymeric products. Pure macromolecular conjugates were obtained in high yield (on average 82% and 79%). The chemical structures of the prepared macromolecular conjugates were confirmed by ^1^H-NMR and FTIR studies. Typical proton NMR spectra of the synthesized products of 6AR/PCL and 6AR/PLLA with coupled ampicillin are shown in Figure 1 and Figure 2.

**Figure 3:**
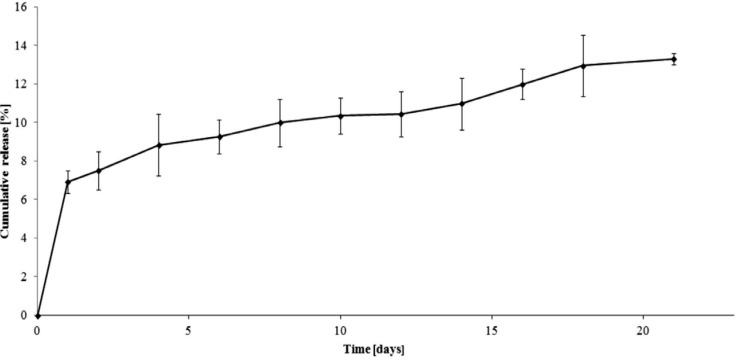
Release profile of ampicillin from the A6AR/PCL/ampicillin conjugate (pH 7.00 ± 0.05).

The spectra of the obtained conjugates are revealed characteristic signals of ampicillin, indicating successful synthesis of the branched polyester-ampicillin conjugates. The formation of an ester bond between the 6AR/PCL and 6AR/PLLA hydroxyl groups and the carboxyl group of antibiotic was demonstrated by an upfield shift in the ^1^H-NMR chemical shift of the proton signal **5** on the thiazolidine ring of ampicillin (Figure 1 and Figure 2) (^1^H-NMR spectra of a pure ampicillin (Figure 1, Supplementary) sample revealed this signal at a downfield shift at 4.18 ppm). Furthermore, the covalent conjugation of ampicillin to the branched polymers was confirmed by the disappearance the signals of the end hydroxyl groups on the spectra of the synthesized 6AR/PCL and 6AR/PLLA samples (presented in our previous paper [19]. In addition, ^1^H-NMR spectra of a pure PCL and PLLA are shown in the Supplementary, denoted as Figure 2 and Figure 3).

The ampicillin content in the 6AR/PCL/ampicillin and 6AR/PLLA/ampicillin conjugates was calculated by integration of the ^1^H-NMR spectra. The signal intensities of two protons on the β-lactam ring of ampicillin (**3** + **4**, Figure 1 and Figure 2) and the signal intensity of the two protons of the -CH_2_- group of PCL (**d**, Figure 1) and the proton of the -CH- group of PLLA (**a**, Figure 2) were compared, indicating the 15.6 and 10.2 mole % drug content in the 6AR/PCL/ampicillin and 6AR/PLLA/ampicillin conjugates, respectively (calculated as mole of the drug per mole of the whole macromolecular system [28]. The one is dependent on the structure of the synthesized branched macromolecules and is increased, when the number of the surface hydroxyl end groups was increased.

### Drug Release from the 6AR/PCL/Ampicillin and 6AR/PLLA/Ampicillin Conjugates

Understanding the drug release characteristics is crucial for the successful design of macromolecular conjugates. The branched macromolecular conjugates of ampicillin obtained in this study have been placed in the buffer solution at pH 7.00 ± 0.05 (phosphate buffer solution) at a temperature of about 37 °C. The drug release study was carried out by 21 days. It allowed assessing the usefulness of macromolecular conjugates for further potential applications (e.g., drug delivery systems or other medicinal products with modified-release).

**Figure 4:**
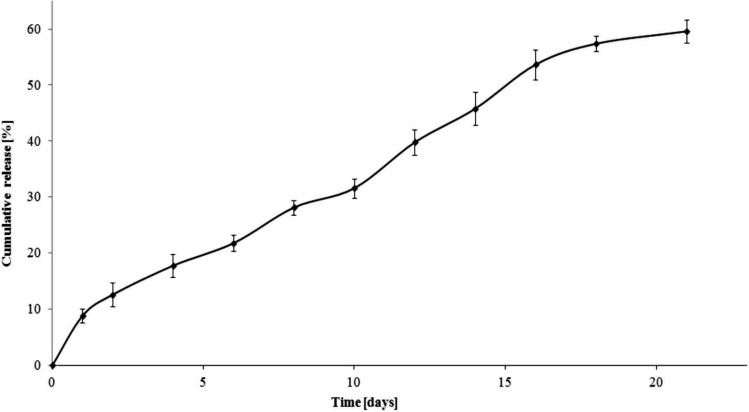
Release profile of ampicillin from the A6AR/PLLA/ampicillin conjugate (pH 7.00 ± 0.05).

**Figure 5:**
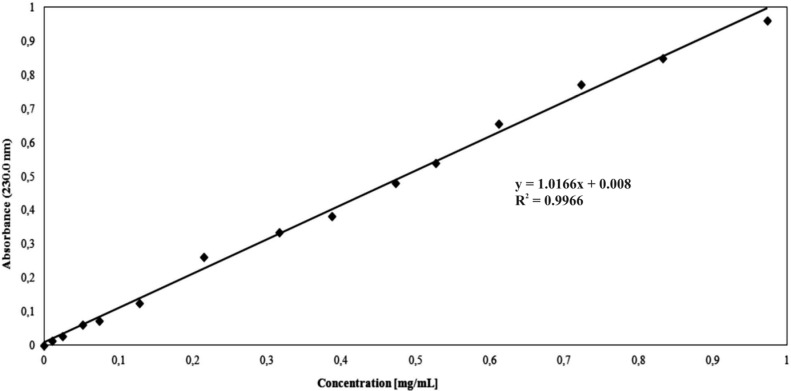
Calibration curve showing the relationship between ampicillin concentration and absorbance in the phosphate buffer solution.

The dependence of the percentage of mass antibiotic released during the time for 6AR/PCL/ampicillin and 6AR/PLLA/ampicillin conjugates are given in Figure 3 and Figure 4. The mass percentage of the released drug was calculated from the calibration curve (Figure 5) at the analytical wavelength λ = 230 nm for the free drug.

The total percentage of the released ampicillin for 6AR/PCL/ampicillin was approximately 14%, while for 6AR/PLLA/ampicillin it was about 60% after 21 days. We assume the above results might be affected by a higher degree of crystallinity and hydrophobicity of PCL *versus* PLA.

The mass percentage of the ampicillin released from the 6AR/PCL/ampicillin sample was about 7% after 1 day. In this case, the drug release profile was characterized by a slow release rate and reached a value of 12% and 13.3% by weight after 16 and 21 days, respectively.

On the other hand, the release profile of ampicillin from the 6AR/PLLA/ampicillin conjugate (Figure 4) was characterized by a greater rate of release. Up to 16 days, 54% of the antibiotic was released. It could be directly shows that on average every 8.6% of the total amount of drug was released, at every 3 days as opposed to the 6AR/PCL/ampicillin sample, when on average 2% of the total amount of ampicillin was released at every 3 days.

The profile of ampicilin release from the matrices based on poly(methylmethacrylate) (PMMA) grafted chitosan was carried out by Chandy and Changerath and coworkers [29]. They demonstrated that 60% of the drug was released after 8 days of incubation for the chitosan-g-PMMA matrix. In our study, almost 10% of the ampicillin was released after 8 days of study for the 6AR/PCL/ampicillin sample, while for 6AR/PLLA/ampicillin the same value was achieved after 1.5 day due to different physico-chemical properties of the synthesized branched polymer matrices.

The release profile of ampicillin sodium salt from the electrospun PCL nanofiber yarns was also studied by Liu and coworkers [16]. The study revealed that nearly 100% of the attached drug was released within 96 h of incubation. Due to the low specific attachment of the drug to the polymeric material, the highest release rate was observed for the first hour of study. The above studies showed that the method of the conjugation of ampicillin to the polymer matrices has a significant impact on the later release profile of the drug. Undoubtedly, the covalent conjugation of the drug to the carrier determines it’s release profile as well as ability for the prediction of the macromolecular conjugate behaviour in a suitable medium, and thus allow to obtain the drug profile release with the intended pharmacokinetics.

In summary, novel, branched macromolecular conjugates of ampicillin have been synthesized and characterized in this study. Natural arginine-6-oligomer has been effectively engaged as an initiator of ROP of cyclic esters forming biodegradable and bioresorbable branched polymer matrices for the covalent conjugation of ampicillin. Furthermore, it’s worthy to emphasize that used initiator is composed of six arginine molecules, exogenous amino acid naturally occurs in the food component (protein) and synthesized in the body. It is involved in the synthesis of biologically important compounds such as nitric oxide, arginine or creatine and also exhibit immunomodulatory and hypotensive effects. The received results are promising to be used in the pharmaceutical technology, e.g., as implantation therapeutic systems.

## 3. Experimental

### 3.1. Materials

Ampicillin ((2*S*,5*R*,6*R*)-6-([(2*R*)-2-amino-2-phenylacetyl]amino)-3,3-dimethyl-7-oxo-4-thia-1-aza- bicyclo[3.2.0]heptane-2-carboxylic acid, d-(−)-α-aminobenzylpenicillin, anhydrous, 96.0%–100.5% (anhydrous basis), Sigma Co. Poznan, Poland), N,N'-dicyclohexylcarbodiimide (DCC, ≥99.0%, Fluka Co. Poznan, Poland) and 4-(dimethylamino)pyridine (DMAP, ≥99%, Aldrich Co. Poznan, Poland) were used without further purification. Dichloromethane (DCM, anhydrous, ≥99.8%, POCh, Gliwice, Poland), dimethyl sulfoxide (DMSO, anhydrous, 99%, Aldrich Co.) and methanol (anhydrous, 99.8%, POCh, Gliwice) were used as received. Phosphate buffer solution (0.1 M, pH 7.00 ± 0.05 (Na_2_HPO_4_-C_6_H_8_O_7_XH_2_O), 20 °C, Chempur, Piekary Slaskie, Poland) was also used as received.

### 3.2. Methods

#### 3.2.1. Branched Macromolecular Conjugate Synthesis

The macromolecular conjugates were prepared under argon atmosphere. The synthesis and physicochemical characterization of the branched biodegradable polyesters (PCL and PLLA) contained arginine-6-oligomer (6AR) as a core and denoted as 6AR/PCL and 6AR/PLLA, have been described in our previous paper [19].

#### 3.2.2. Synthesis of Ampicillin Conjugated 6AR/PCL

Ampicillin (44 mg, 127 µmol), DCC (26 mg, 127 µmol) and DMAP (23 mg, 190 µmol) were dissolved in anhydrous DMSO (4 mL) in a reaction flask and stirred for 2 h at room temperature for the activation of ampicillin. The 6AR/PCL (the molecular weight determined by the viscosity method equaled 17,000 g/mol [19], 90 mg, 5.3 µmol) was dissolved in anhydrous CH_2_Cl_2_ (4 mL) and added dropwise to the reaction mixture. Ampicillin (24 M equivalent) was thus added to the 6AR/PCL polymer. The reaction was stirred for 48 h at a temperature ranging from 25 to 30 °C. After an appropriate time, the reaction mixture was filtered through a filter paper to remove the dicyclohexyl urea (DCU) formed [28] and the resulting product was allowed to dry for 24 h. After this time, the reaction product was dissolved in dry CH_2_Cl_2_ in order to separate the methylene chloride insoluble fraction containing the unreacted drug. Crystallization of the methylene chloride soluble fraction gave an average 98 mg of dry bioconjugation product (6AR/PCL/ampicillin, an average yield of 82% when the conjugate synthesis was carried out in triplicate). ^1^H-NMR of 6AR/PCL/ampicillin (DMSO-*d*_6_, 300 MHz, *δ*_H_, ppm): 1.12 (d, 2 × CH_3_ of ampicillin (**6**), 1.51–2.19 (m, -CH_2_CH_2_C(O)- (**c** + **c**') and -CH_2_CH_2_CH_2_- (**b** + **b**') of PCL, 2.08–1.66 (broad weakly resolved -CH-(NH)-CH_2_CH_2_CH_2_NH-) of 6AR/PCL (**f**)), 2.24–2.33 (m, -CH_2_CH_2_C(O)- of PCL (**a** + **a**')), 3.30 (t, -CH-(NH)-CH_2_CH_2_CH_2_NH-) of 6AR/PCL (**g**)), 4.01–3.94 (t, -CH_2_O- of PCL (**d** + **d**')), 4.44 (broad weakly resolved -CH-(NH)-CH_2_CH_2_CH_2_NH-) of 6AR/PCL (**h**)), 4.77 (-CH-(NH_2_)- of ampicillin (**2**)), 4.99 (broad weakly dissolved, -CH-C(O)- of ampicillin (**5**)), 5.44–5.52 (broad, β–lactam protons ring of ampicillin (**3** + **4**), 7.11–7.52 (m, broad, aromatic protons ring of ampicillin (**1**) and -HNCO- of 6AR/PCL (**e**), Figure 1). FTIR (KBr, cm^−1^): 3440–3080 (υN-H), 2948 (υ_as_CH_2_), 2863 (υ_s_CH_2_), 1785–1778 (υC=O), 1650–1540 (υC=O) and (δN-H), 1570–1515 (υC=C), 1365–1337 (δ_as_CH_3_) and (δ_s_CH_3_), 1293 (υC-O and C-C), 1241 (υ_as_COC), 1191 (υOC-O), 1171 (υ_s_COC), 756 (δN-H).

#### 3.2.3. Synthesis of Ampicillin Conjugated 6AR/PLLA

Ampicillin (45 mg, 128 µmol), DCC (26 mg, 128 µmol) and DMAP (23 mg, 192 µmol) were dissolved in anhydrous DMSO (4 mL) in a reaction flask and stirred for 2 h at room temperature for the activation of ampicillin. 6AR/PLLA (the molecular weight determined by the viscosity method equaled 14,800 g/mol [19], 90 mg, 6.1 µmol) was dissolved in anhydrous CH_2_Cl_2_ (4 mL) and added dropwise to the reaction mixture. Thus 21 M equivalents of ampicillin was added to 6AR/PLLA polymer. The reaction was stirred for 48 h at a temperature ranging from of 25 to 30 °C. After an appropriate time, the reaction mixture was filtered through a filter paper to remove the dicyclohexyl urea (DCU) formed [28] and the resulting product was allowed to dry for 24 h. After this time, the reaction product was dissolved in dry CH_2_Cl_2_ in order to separate the methylene chloride insoluble fraction containing the unreacted drug. Crystallization of the methylene chloride soluble fraction gave an average 99 mg of dry bioconjugation product (6AR/PLLA/ampicillin, an average yield of 79% when the conjugate synthesis was carried out in triplicate). ^1^H-NMR of 6AR/PLLA/ampicillin (DMSO-*d*_6_, 300 MHz, *δ*_H_, ppm): 1.12 (s, 2 × CH_3_ of ampicillin (**6**), 1.22–1.46 (d, -CH_3_ of PLLA (**b** + **b**'), 1.57–1.69 (broad weakly resolved -CH-(NH)-CH_2_CH_2_CH_2_NH-) of 6AR/PLLA (**d**)), 3.47–3.50 (m, -CH-(NH)-CH_2_CH_2_CH_2_NH-) of 6AR/PLA (**e**)), 4.26–4.30 (broad weakly resolved -CH-(NH)-CH_2_CH_2_CH_2_NH-) of 6AR/PLLA (**f**)), 4.73 (-CH-(NH_2_)- of ampicillin (**2**)), 4.90 (broad weakly dissolved, -CH-C(O)- of ampicillin (**5**)), 5.11–5.23 (q, -CH(CH_3_)- of PLLA (**a** + **a**')), 5.44–5.50 (broad, β–lactam protons ring of ampicillin (**3** + **4**), 7.32–7.49 (m, broad, aromatic protons ring of ampicillin (**1**) and -HNCO- of 6AR/PLLA (**c**), Figure 2). FTIR (KBr, cm^−1^): 3450–3130 (υN-H), 2950 (υ_as_CH_2_), 2875 (υ_s_CH_2_), 1770 (υC=O), 1650–1556 (υC=O) and (δN-H), 1580–1510 (υC=C), 1360–1335 (δ_as_CH_3_) and (δ_s_CH_3_), 1293 (υC-O and C-C), 1241 (υ_as_COC), 1191 (υOC-O), 1172 (υ_s_COC), 756 (δN-H).

#### 3.2.4. Toxicity Assays

A Microtox^®^ assay with the luminescent bacteria *Vibrio fischeri* was performed with the lyophilized bacteria purchased from SDI (Newark, DE, USA). The test was performed using disposable glass cuvettes. Samples were incubated at 15 °C for 15 min and the light output of the samples was recorded with a Microtox^®^ M500 analyzer. As a diluent and a control 2% NaCl was used.

Protoxkit F™ is a multigeneration protozoan growth inhibition bioassay with the ciliate *Tetrahymena thermophila* [21,22]. The test is based on the turnover of the substrate (food suspension) into ciliate biomass. While normal proliferating cell cultures clear the substrate suspension in 24 h, inhibited culture growth is reflected by remaining turbidity. The test is based on optical density measurements. The protozoa and the food were obtained from MicroBioTests (Mariakerke (Gent), Belgium). The test was performed in disposable spectrophotometric cuvettes according to the standard operational protocol of the producer. As a diluents and a control deionised water (Milli-Q quality) was used.

A Spirotox test with the protozoan *Spirostomum ambiguum* was performed according to the standard protocol [30,31]. The test was carried out in disposable, polystyrene multiwell plates (24 wells). Ten organisms were added to each well of the multiwell. The samples were incubated in the darkness at 25 °C for 24 h. Afterwards the test responses, *i.e.*, different deformations such as shortening, bending of the cell, *etc.*, and lethal response were observed with the use of dissection microscope (magnification of 10). As a diluents and a control Tyrod solution was used.

Prior to the toxicity test, the materials were pulverized. In the direct contact test, two concentrations of the samples were tested: 1 and 2 mg/mL in Spirotox test, and 0.5 and 1 mg/mL in Protoxkit F™ and Microtox^®^ assays. The tested sample was weighted directly to the test containers and poured with 1 mL of diluent. The test organisms were incubated with the suspension of the tested sample. All the samples were run in triplicate for the toxicity measurements.

#### 3.2.5. The *Umu*-Test

The *umu*-test is a short-term bacterial test for genotoxicity assessment of environmental samples as well as chemicals. The test employs *Salmonella typhimurium* TA1535 strain. It was carried out in 96-well microplates with and without the metabolic activation by s9 fraction according to the ISO guideline [32]. S9 fraction was prepared from liver of male Sprague-Dawley rats pretreated with Aroclor 1254 (500 mg/kg) 5 days prior to isolation. The test strain is able to response to different types of DNA damages and therefore to detect different kinds of genotoxins. Genotoxic potential of the sample is measured as the induction of the umuC gene, which is included in the SOS system in bacterial cell. The test strain is genetically changed and as the umuC gene is fused with the lacZ gene (structural gene for β-galactosidase) and additionally the normal lacZ region is deleted, the induction of umuC gene is assessed as the determination of β-galactosidase activity in a simple colorimetric assay. Induction ratio (IR) is calculated to show the genotoxic potency of the tested sample, as the ratio of β-galactosidase activity evaluated for the sample to the negative control. IR ≥ 1.5 is considered as the threshold at which the sample demonstrated the genotoxic activity. The assay is quantitative and the dose-response curves present a linear region. Additionally, the growth factor (G) of bacteria is calculated to verify the acceptable level of cytotoxicity of the samples.

The tested polymers were incubated for 24 h, 37 °C in buffer solution (10 mg/mL) and such an extract was investigated in the test. Additionally 1 mg of the tested polymers was placed directly in the microplate and incubated with bacteria. The exposure of the bacterial strain to the tested samples was carried out in both cases for 2 h, 37 °C. Deionized sterile water was used as a negative control and 4-nitroquinoline-*N*-oxide and 2-aminoanthracene as positive controls.

#### 3.2.6. *In Vitro* Ampicillin Release Studies

The *in vitro* release study of ampicillin from the synthesized branched polyesters was investigated by measuring the concentration of ampicillin released at pH 7.00 ± 0.05 (0.1 M phosphate buffer solution). All experiments were carried out in triplicate. Dried 6AR/PCL/ampicillin and 6AR/PLLA/ampicillin macromolecular conjugate (90 mg), respectively, were immersed into buffer solution (90 mL, pH 7.00 ± 0.05) and incubated at 37 °C, with continuous orbital rotation at 50 [cycles/min]. At predetermined time intervals, 5 mL samples were withdrawn from the release medium using the filter followed by replace with 5 mL of a fresh buffer solution. The products were separated by centrifugation (10,000 rpm). The absorption of buffer solution was determined by a UV-Vis spectrophotometer at the absorbance peak with a wavelength at 230 nm. The absorbance peak were correlated with the concentration of ampicillin released. As shown in Figure 5, a linear calibration curve was obtained by measuring the solutions absorption with ampicillin concentrations. For all the measurements in this study, the absorbance readings were within the calibration range [5].

#### 3.2.7. Characterization Techniques

The polymerization products were characterized in the DMSO-d_6_ solution by means of ^1^H-NMR (Varian 300 MHz). The FTIR spectra were measured from KBr pellets (Perkin - Elmer spectrometer).

The amount of the released ampicillin was quantitatively determined by a UV-Vis spectrophotometry (UV-1202 Shimadzu) in aqueous buffered solutions at the adsorption maximum of the free drug (*λ* = 230 nm) using a 1 cm quartz cell.

## 4. Conclusions

Novel 6AR/PCL/ampicillin and 6AR/PLLA/ampicillin macromolecular conjugates have been obtained by the conjugation of the β-lactam antibiotic to the synthesized branched polymeric matrices, based on a natural oligopeptide core. The presence of a newly formed ester bond between the drug and the polymer matrices was confirmed by ^1^H-NMR and FTIR studies. The obtained macromolecular products have been subjected to further *in vitro* release studies as well as cyto- and genotoxicity in order to verify their potential usefulness as polymeric drug carriers.

The total percentage of ampicillin released after 21 days of incubation was nearly 60% and 14% for the 6AR/PLLA/ampicillin and 6AR/PCL/ampicillin samples, respectively. This significantly different antibiotic profile release resulted from the different physicochemical properties of the polymeric matrices.

The obtained results showed that the conjugation method of the drug through an ester linkage results in a more stable macromolecular conjugate form, thus the obtained matrices allowed for a controlled drug release profile and might improve the pharmacokinetics of a pharmacologically active substance. The results are promising for applying the obtained macromolecular conjugates as implants (medium or long-term) in pharmaceutical technology.

## Supplementary Materials

Supplementary materials can be accessed at: http://www.mdpi.com/1420-3049/19/6/7543/s1.
