# Supplementary material for: Ampicillin-Ester Bonded Branched Polymers: Characterization, Cyto-, Genotoxicity and Controlled Drug-Release Behaviour
Source: Molecules. 2014 Jun 6;19(6):7543–56. doi: 10.3390/molecules19067543 (PMC6271874; doi:10.3390/molecules19067543)

Supplementary Information

Figure S1. ^{1}H-NMR spectrum of ampicillin.

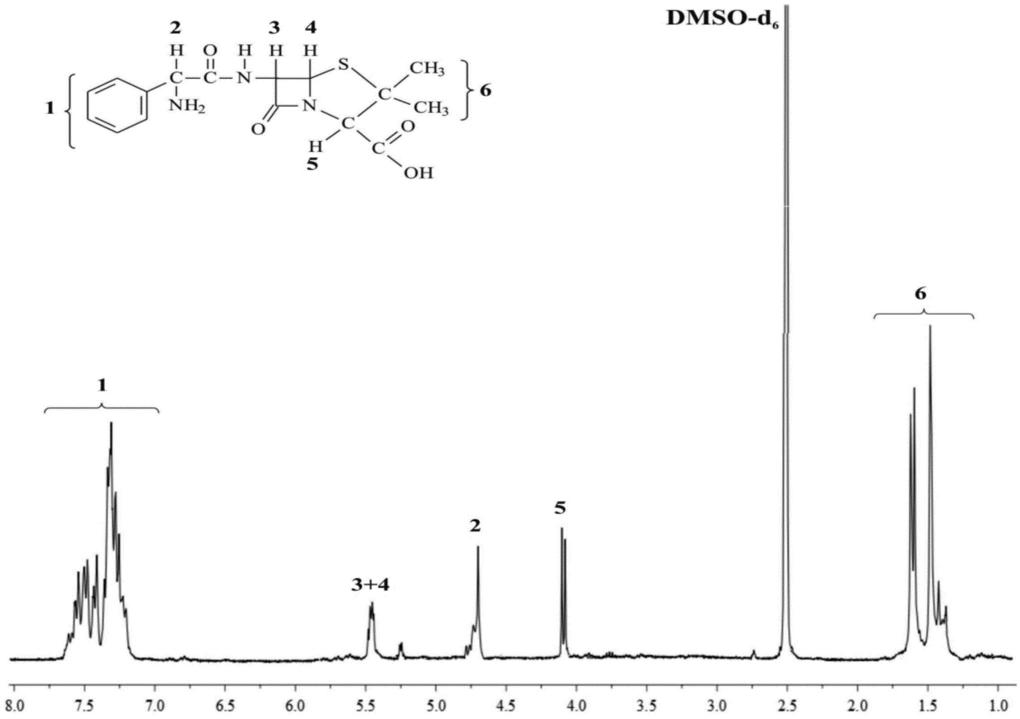

Figure S2. ^{1}H-NMR spectrum of poly(ε-caprolactone) (PCL).

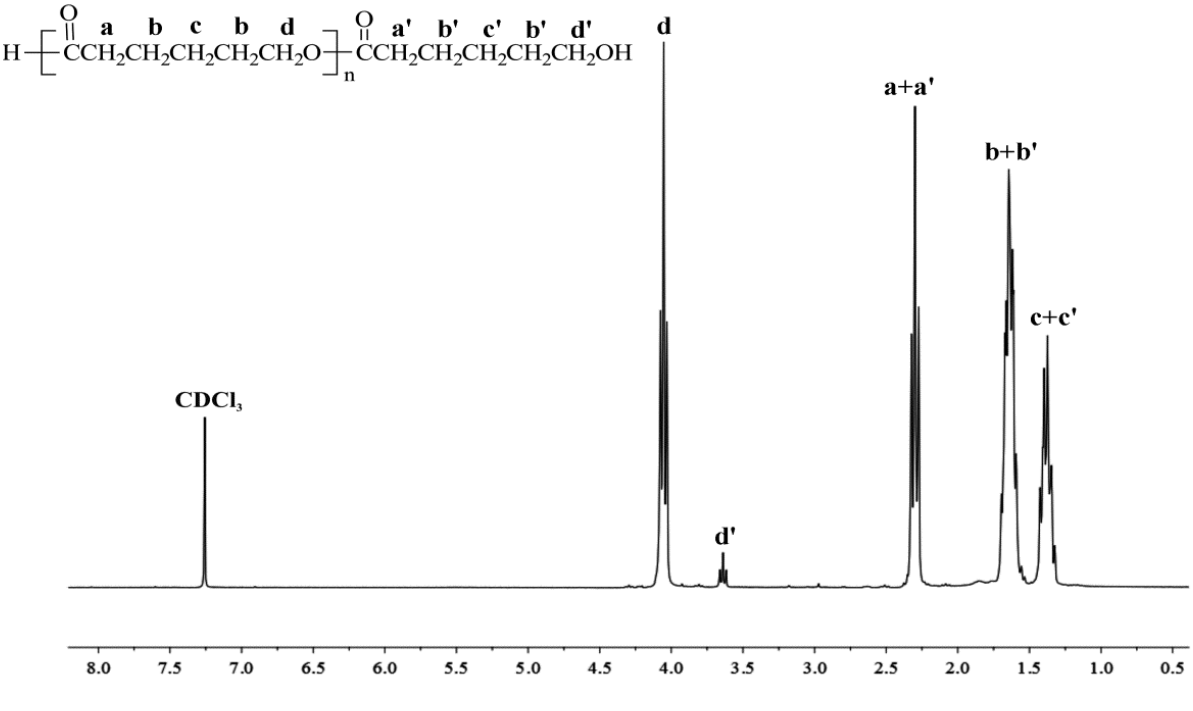

Figure S3. ^{1}H-NMR spectrum of poly(L,L-lactide) (PLLA).

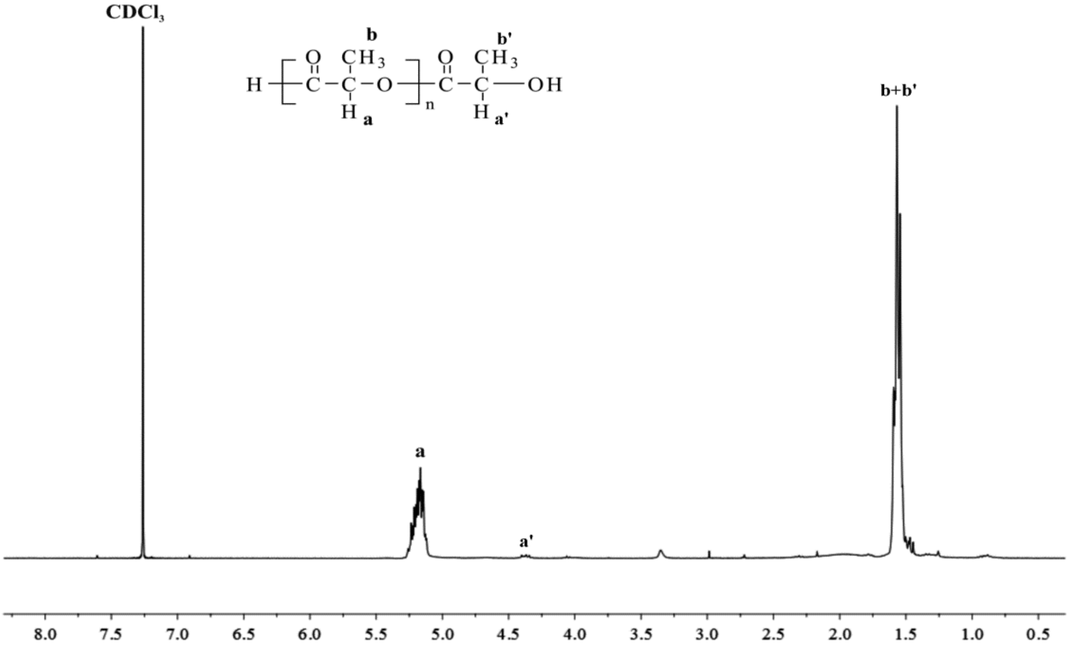

Supplement: Supplementary file 1 [file molecules-19-07543-s001.pdf]
